# The potential of antisense oligonucleotide therapies for inherited childhood lung diseases

**DOI:** 10.1186/s40348-018-0081-6

**Published:** 2018-02-06

**Authors:** Kelly M. Martinovich, Nicole C. Shaw, Anthony Kicic, André Schultz, Sue Fletcher, Steve D. Wilton, Stephen M. Stick

**Affiliations:** 1 School of Paediatrics and Child Health, The University of Western Australia, Nedlands, Western Australia 6009 Australia; 2 Telethon Kids Institute, Centre for Health Research, The University of Western Australia, Nedlands, Western Australia 6009 Australia; 3 Centre for Cell Therapy and Regenerative Medicine, School of Medicine and Pharmacology, The University of Western Australia, Nedlands, Western Australia 6009 Australia; 4 Department of Respiratory Medicine, Princess Margaret Hospital for Children, Subiaco, Western Australia 6008 Australia; 5 School of Public Health, Curtin University, Bentley, Western Australia 6102 Australia; 6 Perron Institute for Neurological and Translational Sciences, The University of Western Australia, Nedlands, Western Australia 6009 Australia; 7 Centre for Comparative Genomics, Murdoch University, Murdoch, Western Australia 6150 Australia

**Keywords:** Antisense oligonucleotides, Cystic fibrosis, Surfactant disorders, Childhood, Inherited diseases

## Abstract

Antisense oligonucleotides are an emerging therapeutic option to treat diseases with known genetic origin. In the age of personalised medicines, antisense oligonucleotides can sometimes be designed to target and bypass or overcome a patient’s genetic mutation, in particular those lesions that compromise normal pre-mRNA processing. Antisense oligonucleotides can alter gene expression through a variety of mechanisms as determined by the chemistry and antisense oligomer design. Through targeting the pre-mRNA, antisense oligonucleotides can alter splicing and induce a specific spliceoform or disrupt the reading frame, target an RNA transcript for degradation through RNaseH activation, block ribosome initiation of protein translation or disrupt miRNA function. The recent accelerated approval of eteplirsen (renamed *Exondys 51*™) by the Food and Drug Administration, for the treatment of Duchenne muscular dystrophy, and nusinersen, for the treatment of spinal muscular atrophy, herald a new and exciting era in splice-switching antisense oligonucleotide applications to treat inherited diseases. This review considers the potential of antisense oligonucleotides to treat inherited lung diseases of childhood with a focus on cystic fibrosis and disorders of surfactant protein metabolism.

## Introduction

Antisense oligonucleotides (AOs) have the ability to modulate gene expression by interacting with specific gene transcripts through a variety of mechanisms. Decades ago, ambitious claims were made regarding the potential of AO technology to modulate gene expression in the treatment of human disease, but with most new drug discovery platforms, various factors including inconsistent synthesis protocols, inadequate understanding of mechanisms and limited experimental controls have hindered the technology’s progress. Only a handful of AOs have been approved for clinical use to date, despite years of clinical development, and in hindsight, it is remarkable that each approved treatment invokes a different mechanism of action [1].

Recently, two new drugs have been approved to treat two “common” rare and severe genetic diseases: Duchenne muscular dystrophy (DMD) [2] and spinal muscular atrophy (SMA) [3]. In both these cases, the intervention is designed to redirect pre-mRNA processing, in the case of DMD, through induction of targeted exon skipping, while in SMA, the aim is to promote exon retention to produce a functional transcript. All human genes undergo some form of pre-mRNA processing during expression and the majority of primary gene transcripts undergo either constitutive or alternative splicing, where noncoding intronic sequences are removed from the mature mRNA and exons are precisely spliced together. Mutations that cause abnormal splicing, complete or partial exon loss or the retention of intronic sequences, are now recognised as being relatively common causes of human disease [4]. Hence, the potential for AO modification of gene expression as a therapeutic strategy is currently being explored in other disease settings.

Vigorous research is focussing on refining AO chemistries and modifications to improve bioavailability, safety, potency and reduction of off-target effects [5–7]. However, challenges facing the clinical translation of this technology still exist, particularly with respect to efficient delivery and nuclear uptake of AO in target tissues, and will vary depending upon the target tissue and disease. In addition, clinical trial design and evaluation of novel therapeutics in conditions where the patient population is small and disease progression is slow will most likely demand innovative and adaptive trials [8–11]. In this review, we provide an introductory overview of AO chemistries, mechanisms and the advantages of each with particular attention to the potential to treat respiratory diseases of genetic origin.

## Review

### Antisense oligonucleotide chemistry

Antisense oligonucleotides are short, single strands of nucleic acid, commonly cited to be 13–50 nucleotides long but in practise are more typically 20–25 bases in length. Appropriately designed compounds can bind with high affinity and specificity to given nucleic acid targets through Watson and Crick base-pairing [12]. As the natural phosphodiester backbone of RNA and DNA is particularly susceptible to nuclease degradation, the chemistry used for each nucleotide in early studies that used AOs was unstable and generally ineffective. This limitation was quickly recognised, and chemical modifications to the oligonucleotide backbone were made, aiming to improve target affinity, nuclease resistance, toxicology profile, biostability and pharmacokinetics [13–21]. To date, a wide variety of AO analogues have been generated that vary in their pharmacological properties, and these further contribute to the mechanisms of action [5]. The chemistry of particular AO analogues have been reviewed in depth elsewhere [15–17] and will not be discussed in detail in this review. Properties of commonly used nucleotide chemistries that are used to formulate AOs are summarised in Table 1.

**Table 1:** Common nucleotide chemistries used to synthesise antisense oligonucleotide molecule

Nucleotide chemistry	Abbreviation	Resistant to nuclease degradation	RNase H activation	Splice modulation	Toxicity	Charge	Reference
Phosphorothioate DNA	PS	+	+	–	++	Negative	[15]
2′-*O*-methyl RNA	OMe	+	–	+	+	Negative	[16]
2′-*O*-Methoxyethyl RNA	MOE	+	–	+	–	Negative	[13, 17]
Locked nucleic acid	LNA	+	–	+	+	Uncharged	[14, 18, 19]
Phosphoroamidate morpholino	PMO	+	–	+	–	Uncharged	[20]
Peptide nucleic acid	PNA	+	–	+	–	Uncharged	[21]

As mentioned above, AOs can modulate gene expression through a variety of mechanisms depending on the base modification, backbone and sequence design. Some AO chemistries activate RNase H, a ubiquitous ribonuclease, which specifically hydrolyses the phosphodiester bonds of the RNA strand in DNA to RNA hybrids, thereby degrading the RNA transcript and preventing translation of the encoded protein. RNase H activity is supported by DNA-like AO chemistries on a phosphorothioate backbone [18]. Although the inclusion of the phosphorothioate backbone does confer some increased nuclease resistance to the AOs, these compounds have a limited biological half-life of around 35 to 50 h [19]. Extended AO stability and hence activity has been achieved through the incorporation of modified bases, such as LNA, 2′-*O*-methyl and 2′-*O*-methoxyethyl modifications at the 5′ and 3′ ends of the oligomer to make a “gapmer” [5]. Other AO-induced mechanisms of gene downregulation may involve the steric inhibition of ribosomal complex formation by preventing translation initiation, modulating the pre-mRNA splicing process to disrupt the reading frame or by removing exons encoding crucial functional domains [20]. 2′-*O*-methyl- or 2′-*O*-methoxyethyl-modified oligonucleotides, peptide nucleic acids (PNAs) and phosphorodiamidate morpholino oligomers (PMOs) do not support RNase H induction and are also potential steric blocking compounds.

More than 95% of human genes consist of introns and exons and must undergo splicing during pre-mRNA maturation. At least 75% of our genes undergo some degree of alternative splicing to generate multiple isoforms from one gene. Hence, it is possible that the application of AOs to treat a wide variety of human gene mutations lies in therapeutic alternative splicing. Through re-directing the normal pre-mRNA splicing process, AOs could potentially generate non-functional transcripts by targeted excision of a frameshifting exon that would then render the induced transcript susceptible to degradation through the nonsense-mediated decay pathway [21]. Where some splice mutations result in non-functional or deleterious protein isoforms, AOs could be targeted to mutated splice motifs, thereby modulating the splicing process and restoring the expression of functional protein. While intra-exonic and intronic mutations that affect splicing enhancers could potentially be targeted by AOs, intronic mutations that involve the invariant bases flanking exons are unlikely to be amenable to AO therapy. Splice-switching antisense oligomers can also restore the function of a disease-causing allele by altering a splice site or exon selection to restore the open reading frame, compromised by a disease-causing mutation.

### Use of antisense therapy in disease

**Fig. 1:**
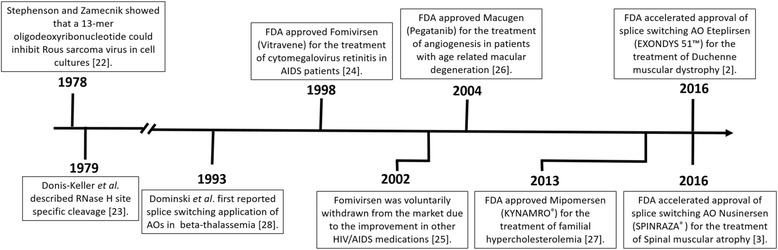
Milestone timeline of the development and clinical use of antisense oligonucleotides

In 1978, Stephenson and Zamecnik showed that a 13-mer oligodeoxyribonucleotide could inhibit Rous sarcoma virus in cell cultures [22], and RNase H site-specific cleavage was described soon after [23] (Fig. 1). However, it was not until 1998 that the first approved use of AOs occurred, when the Food and Drug Administration permitted the commercialisation of fomivirsen (Vitravene) for the treatment of cytomegalovirus retinitis in AIDS patients [24]. Fomivirsen is a 21-mer phosphorothioate oligodeoxyribonucleotide that is complementary to a portion of the human cytomegalovirus (HCMV) gene and inhibits HCMV replication through an antisense mechanism. However, due to the improvement in other HIV/AIDS medications leading to the decline in HIV-associated opportunistic CMV infections at the time, Fomivirsen was voluntarily withdrawn from the market in 2002 [25]. The next FDA-approved use of antisense oligonucleotides was Macugen (pegatanib). Macugen is a single-stranded nucleic acid aptamer that binds to the heparin-binding domain of vascular endothelial growth factor (VEGF)-165 with extremely high affinity [26]. VEGF is involved in pathologic angiogenesis; in particular, VEGF-165 promotes angiogenesis in the eyes of patients with age-related macular degeneration. In 2004, Macugen was approved as a treatment for age-related macular degeneration. In 2013, mipomersen (KYNAMRO®) was approved by the FDA for the management of homozygous familial hypercholesterolemia, a genetic disorder characterised by high cholesterol [27]. Mipomersen is an oligonucleotide inhibiter of Apolipoprotein (Apo) B-100 synthesis. The AO sequence is complementary to the Apo B-100 messenger RNA and, when bound, activates RNase-H enzymatic cleavage of the mRNA, thereby inhibiting translation of the gene product and reducing circulating low-density lipoproteins that contribute to familial hypercholesterolemia disease [27].

The above examples use AOs to alter gene expression, either directly or via competitive binding to receptors, to change disease progression. Another precise method to alter disease progression is to manipulate splicing of exons within a gene transcript using splice-switching AOs. The first reported example of AO-mediated splice-switching was reported by Dominski and Kole in 1993 who used AOs to correct abnormal splicing of a β-globin transcript responsible for beta-thalassemia. This was originally done in vitro with vector constructs and cell-free extracts [28] and later in peripheral blood cells [29] but has not yet proceeded to clinical trials. Therapeutic interventions that exploit alternative splicing are also being examined in diseases such as angioplasty restenosis [30], cancer [31, 32], amyotrophic lateral sclerosis [7, 33] and Huntington’s disease [34].

**Fig. 2:**
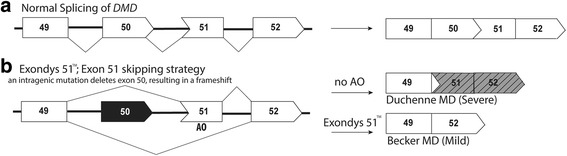
Exondys 51™ excludes dystrophin exon 51 and corrects the *DMD* reading frame, therefore reducing disease severity in the most common type of DMD mutation. **a** Normal splicing of *DMD* exons 49–52. **b** Exon 51 is deleted from the *DMD* transcript as the result of a mutation at affect about 13% of DMD patients. Exondys 51 alters splicing of exon 51, removing it from the *DMD* transcript, restoring the reading frame and reducing disease severity, reflecting a Becker MD phenotype

DMD is an X-linked recessive muscle-wasting disease caused by mutations in the *DMD* gene that lead to premature termination of translation [35]. A milder form of disease, Becker muscular dystrophy (BMD), is generally caused by in-frame deletions in the dystrophin gene that lead to an internally truncated dystrophin protein that retains partial function. The rationale behind splice-switching therapy for DMD is to redirect dystrophin pre-mRNA processing and induce a BMD-like dystrophin mRNA that would be translated into a protein that retains some level of function and thereby reduce disease severity. Exondys 51 is specifically designed to address the most common sub-group of DMD mutations, selected genomic deletions flanking exon 51, and will be relevant to approximately 13% of DMD deletion patients (Fig. 2). Boys that have been receiving Exondys 51 are remaining ambulant and have reduced respiratory muscle decline when compared to historical data [36]. Recent phase 1/2 clinical trials with golodirsen developed to skip exon 53 in DMD have shown a 10-fold increase in dystrophin protein expression in muscle samples after 48 weeks of treatment [37], with phase 3 trials ongoing (NCT02500381).

Different DMD-causing mutations require other splice-switching strategies to reframe the dystrophin mRNA, and at least five additional compounds targeting exons 51, 53, 46, 50 and 43 would address up to 43% of DMD mutations [38]. To further increase the spectrum of mutations that can be targeted, multiple AOs could be combined as a cocktail to target blocks of sequential exons [39]. In addition to the increased range of mutations that can be targeted, successful multiple exon skipping combinations can then be applied to a broader pool of patients. This strategy has been developed for several mutations in DMD but is yet to be translated [39–41]. It has also been proposed that skipping exons 45–55 of the *DMD* gene could result in a functional protein isoform and rescue up to 63% of patients with DMD [42].

**Fig. 3:**
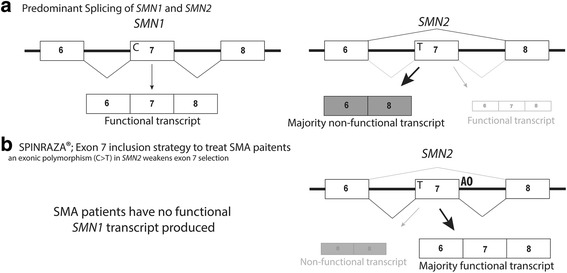
SPINRAZA® strengthens exon 7 recognition and retention in the *SMN2* mRNA transcript, reducing SMA disease severity. **a** Predominant splicing of *SMN2* exons 6–9. **b** An exonic polymorphism weakens exon 7 selection in the *SMN2* mRNA. SPINRAZA® strengthens exon 7 selection in the *SMN2* transcript, producing a functional protein and reducing SMA disease severity

SMA is a fatal autosomal recessive neuromuscular disease that results in progressive paralysis with muscular atrophy in the first years of life [43]. Survival motor neuron (*SMN*) 1 and *SMN2* genes encode the SMN protein, and although both genes potentially code for identical proteins, a C>T polymorphism in the third base of a glycine codon in exon 7 disrupts normal processing of *SMN2*, such that exon 7 is omitted from the majority of the transcripts. SMA is caused by the homozygous loss of the *SMN1* gene and inability of two copies of *SMN2* to compensate protein production due to inefficient splicing of exon 7 [44]. A splice-switching AO was developed to target the *SMN2* intronic splice silencer element ISS-N1 and promote exon 7 inclusion, thereby allowing *SMN2* to produce the full-length and functional SMA protein [45] (Fig. 3). In 2016, the splice-switching AO therapeutic, nusinersen (SPINRAZA®), was approved by the FDA [46]. Nusinersen is injected intrathecally in patients as soon as a SMA diagnosis is confirmed. Nusinersen increases full-length SMN protein levels in the spinal cord, therefore improving motor function in SMA patients and presumably extending life expectancy [47].

### Potential of antisense oligonucleotide therapy in CF

Cystic fibrosis (CF) is a life-threatening, autosomal recessive genetic disease that affects multiple organs, caused by mutations in the gene encoding the CF transmembrane conductance regulator (CFTR) protein. These mutations result in either a complete lack or reduction of protein or an incorrect functioning protein at the apical surface of epithelial cells [48]. The predominant clinical effects of *CFTR* mutations are progressive lung disease and malnutrition. In the lungs, abnormal function of chloride channels results in a dehydrated airway surface. Poorly functioning mucociliary clearance leads to chronic inflammation, infection and progressive destruction of lung tissue, followed by respiratory failure [49]. In the pancreas, pancreatic duct obstruction results in failure of pancreatic exocrine function, in particular the loss of lipase activity, causing fat malabsorption. Whereas the nutritional consequences of CFTR dysfunction can be effectively treated by exogenous pancreas and diet, the pulmonary consequences have proved less easy to treat. Mutations have been identified in all *CFTR* exons and introns [50], grouped into six classes of *CFTR* mutations that have already been extensively reviewed [49, 51]. The biological consequences of *CFTR* mutations are numerous, and two main types of drugs have been investigated to correct these effects: the potentiators and correctors that increase ion flow (class III and IV mutations) and enhance productive trafficking (class II mutations), respectively [52, 53].

Over 2000 known mutations of *CFTR* causing CF have been identified, but only four, in addition to the most common p.Phe508del that accounts for over 70% of mutations in Caucasians, have frequencies of greater than 1–3%. Multiple drugs are being developed aiming to improve the symptoms of CF including poor mucociliary clearance, inflammation, infection and nutritional deficits. Two approved drugs are currently available to restore CFTR function: ivacaftor (Kalydeco®) and lumacaftor + ivacaftor (Orkambi®). Ivacaftor has been shown to improve the ability of the defective CFTR channel to open, once on the apical surface of the cell [53], and is approved for 33 different mutations. Lumacaftor improves processing of the CFTR protein and transport to the apical surface of the cell [54]. Lumacaftor has been combined with ivacaftor as Orkambi® and is approved for patients homozygous for the p.Phe508del mutation. A summary of CFTR drugs that are currently in phase 2/3 clinical trials is outlined in Table 2. A phase 3 trial of ataluren (Translarna™), a small molecule developed to induce “read-through” of nonsense mutations, commenced in 2015 but was terminated after it failed to improve lung function over 48 weeks of treatment [55]. Of the 2000+ *CFTR* mutations identified, only 33 have an approved and available corrective therapy.

**Table 2:** CFTR drugs in current phase 2 or 3 clinical trials

Name	Clinical trial ID	Co-treatment	Phase	Trial status	Mutation	Comments
GLPG2222	NCT03119649 NCT03045523	– Ivacaftor	2 2	Recruiting Recruiting	508/508 508/class III	Corrector
Cavosonstat (N91115)	NCT02589236 NCT02724527	Orkambi Ivacaftor	2 2	Complete Ongoing, but not recruiting	508/508 508/class III	Increase levels of a signalling molecule S-nitrosogluthione (GSNO)
Tezacaftor (VX-661)	NCT02392234 NCT03150719 NCT02951182 NCT02951195	Ivacaftor Ivacaftor Ivacaftor + VX-440 Ivacaftor + VX-152	3 3 2 2	Complete Recruiting Recruiting Recruiting	508/RF 508/508 508/508; 508/MF 508/508; 508/MF	Move the defective CFTR protein to the proper place in the airway cell surface
CTP-656	NCT02971839	–	2	Recruiting	Gating mutations	Altered form of ivacaftor
Riociguat (BAY63-2521)	NCT02170025	–	2	Recruiting	508/508	Stimulates soluble guanylyl cyclases (sGC), an enzyme in the cardiopulmonary system
QR-010	NCT02532764	–	1/2	Recruiting	508/508	RNA-based oligonucleotide

Data is correct as of 19/07/2017 from https://clinicaltrials.gov/ct2/home

*MF* minimal CFTR function mutation, *RF* residual CFTR function mutation, *508* p.Phe508del

A potential therapeutic strategy to reduce the CF disease burden is to identify which of the ~ 2000 CF mutations are amenable to AO-mediated splice intervention. A study by Sterne-Weiler and colleagues (2014) found that approximately 25% of all pathogenic missense or nonsense mutations actually caused abnormal splicing [4]. If such statistics were applicable to *CFTR* mutations, then up to 500 different mutations may be amenable to AO splice-switching as personalised therapies. Here, manipulation of gene expression using AOs could be used to target specific but less frequent *CFTR* mutations. In CF, nonsense, splicing and frameshift mutations are known to occur in all *CFTR* exons [50] and currently have no effective CFTR function-correcting treatment. AO therapy could be used in specific CF cases to alter splicing to skip an exon containing a nonsense or frameshift mutation, restoring the reading frame, assuming the resultant isoform retained better function than the mutated protein. Such a strategy could restore function to the resulting CFTR protein and reduce the severity of disease in amenable patients. In 2004, Zamecnik et al. constructed a modified oligodeoxyribonucleotide to restore CFTR function by inserting into the mRNA the three missing bases caused by the p.Phe508del CFTR mutation. The AO had a central segment containing a phosphorothioate modification and the adjoining segments phosphorothioate plus 2′-*O*-methyl modifications [56]. This modified AO was used in vitro and showed improved CFTR function [56]. There is currently a phase 1/2 clinical trial ongoing using a modified RNA oligonucleotide QR-010 that is designed for p.Phe508del mutation and is based on the 2004 publication by Zamecnik and colleagues [55].

**Fig. 4:**
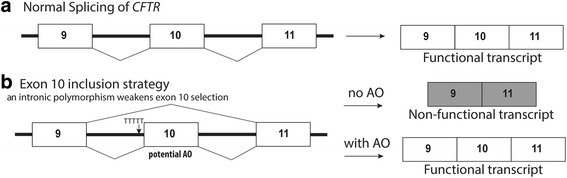
Deleterious alternative splicing of *CFTR* exon 10 could be addressed using splice-switching AOs. **a** Normal splicing of exons 9–11 of *CFTR*. **b** An intronic polymorphism weakens exon 10 selection in *CFTR* mRNA. AO-mediated retention of *CFTR* exon 10 in the mature mRNA

In addition to exon skipping strategies, a proportion of sequence variations and synonymous changes could weaken splicing, compromising exon selection and retention in the mature mRNA and thereby contribute to disease severity. Deleterious alternative splicing of *CFTR* exon 10, mediated by intronic polymorphisms, has been reported as a cause of CF in a subset of patients [57–59]. AOs can be used to strengthen the retention of *CFTR* exon 10 in the mature mRNA and could influence the manifestation of the disease in patients with various mutations, or used to complement alternative strategies by increasing the mRNA levels (Fig. 4). An in vitro example is for CFTR splicing mutation 2657+5G>A mutation that causes exon 16 to be excluded during splicing; however, the transcript was corrected by an AO designed to strengthen exon 16 inclusion [60]. A mutation found in 5% of Ashkenazi Jewish patients, 3849 + 10 kb C → T, creates a novel donor site in intron 19, causing an 84 base-pair pseudo-exon to be included in the mRNA and generation of a premature stop codon [61–63]. In vitro correction of this splicing defect using AOs was achieved in 1999 by Friedman et al.; however, no clinical application is yet available [62].

### Antisense oligonucleotide therapy for the treatment of disorders of surfactant protein metabolism

Pulmonary surfactant, produced by type II alveolar cells in the alveoli, is critical to overcome surface tension at the air-liquid interface and prevent alveolar collapse upon end expiration [64]. Lamellar bodies, present in type II alveolar cells, are the key subcellular organelles involved in surfactant metabolism. Pathogenic mutations in genes involved in surfactant production and homeostasis have been associated with neonatal respiratory distress in infants and diffuse lung disease in children [65, 66]. Collectively, inherited conditions caused by pathogenic mutations in the genes encoding surfactant protein B (*SFTPB*), surfactant protein C (*SFTPC*) and ATP-binding cassette subfamily A member 3 (*ABCA3*) are referred to as disorders of surfactant protein metabolism (DSPM). Conditions associated with *SFTPB* and *ABCA3* mutations demonstrate autosomal recessive inheritance, while *SFTPC* disorders are autosomal dominant. Although rare, DSPM are associated with high morbidity and mortality, with a spectrum of pathology/phenotype that ranges from rapidly fatal in the neonatal period to chronic lung disease that slowly resolves during childhood [67]. Surviving patients require considerable healthcare input in the first years of life and often require extended stays in hospital. Age of disease onset, histopathology of lung tissue, lamellar body ultrastructure and clinical outcomes vary considerably according to the gene affected and have been reviewed elsewhere [65, 66, 68].

No mechanism-specific interventions are currently available to treat DSPM, and treatment options are limited to supportive care, use of drugs that may confer serious long-term side effects or lung transplantation to prolong survival [69]. Clinical presentation of neonatal respiratory insufficiency is similar to respiratory distress syndrome of prematurity, yet treatment with exogenous surfactant has been shown to have little benefit in patients with DSPM [70]. Systemic corticosteroids are often prescribed empirically to treat diffuse lung disease associated with *ABCA3* or *SFTPC* mutations by immunomodulation, but clinical trial data that demonstrates efficacy of this therapeutic strategy is lacking at this time. Some case reports do support the use of hydroxychloroquine for patients presenting with diffuse lung disease [71], while other reports propose a combination of intravenous pulse methylprednisolone and azithromycin with hydroxychloroquine [72], but these treatment regimens may only be beneficial in a subset of patients. Moreover, concerns regarding the risks associated with long-term use of antibiotics, corticosteroids [73] and hydroxychloroquine [70] are well documented. Therefore, there is a need to develop new therapeutics that directly target the disease mechanism.

**Fig. 5:**
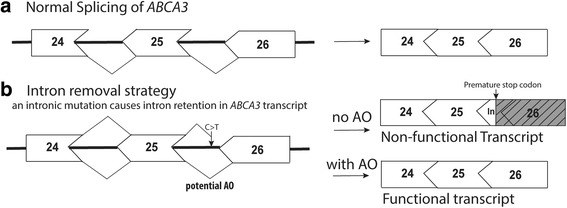
Splice mutation in *ABCA3* causes partial intron 25 inclusion in the mRNA resulting in DSPM. **a** Normal splicing of ABCA3 exons 24–26. **b** Aberrant splicing of intron 25 caused by point mutation, IVS-98T, introduces a stop codon after 77 additional amino acids after exon 25, resulting in a truncated protein. AO-mediated splice correction could potentially reduce the disease severity

The use of AOs to treat disorders of surfactant protein metabolism is one therapeutic approach that has yet to be explored. A vast number of pathogenic mutations including frameshifting indel, nonsense, missense and splice mutations in surfactant-related genes have been identified to date [65, 74]. Depending on the nature of the mutation, a range of AO splice-correcting strategies could be exploited in an attempt to restore protein function and attenuate disease. AOs could sterically block aberrant splice sites to inhibit abnormal splicing. A number of splice mutations in *ABCA3* have been reported to date, such as c.1112-20 G>A, c.1742-10 T>A, c.3862+4 A>T and IVS-98T (Fig. 5) [74, 75], that may be amenable to AO therapy. Of the *ABCA3* splice mutations reported in the literature, the effects of specific mutations on exon selection are not often described. While splice mutations in the two invariant intronic bases flanking each exon have also been reported (e.g. c.4909+1 G>A, c.1612-2 A>G), these mutations are not amenable to splice correction, since the canonical splice sites are lost. Alternatively, for disease associated with loss of function of ABCA-3, SP-B or SP-C proteins, AOs could be used to skip exons containing frameshift or nonsense mutations to restore the open reading frame.

The majority of disease-causing mutations in *SFTPC* are found within the BRICHOS domain (exons 4 and 5) of the SP-C pro-peptide, including the first reported *SFTPC* mutation, c.460+1 G, which results in the skipping of exon 4 due to loss of the canonical donor splice site [76]. Mutations in the BRICHOS domain of SP-C result in protein misfolding and cytosolic accumulation of the aberrant protein, with a dominant negative effect [77]. For disease linked to toxic gain-of-function associated with certain *SFTPC* mutations [78], AOs may potentially be utilised to reduce levels of abnormal SP-C proteins, if allele-specific silencing was possible.

### Delivery of antisense oligonucleotide therapy

One of the major challenges in developing AO-based therapeutics is to achieve efficient delivery to disease-relevant tissue in vivo and, in addition, overcome the cellular barriers that prevent AOs from reaching intracellular molecular targets in the nucleus where splicing takes place. Additionally, AO intervention only allows for transient correction of aberrant gene expression or splicing, making the development of appropriate dosing and drug administration regimens paramount.

The route of drug administration is a significant factor influencing the biodistribution of a particular AO. Systemic administration by parental injection (either intravenous infusion or intraperitoneal or subcutaneous injection) typically results in the highest accumulation levels of AO in the liver and kidney followed by the bone marrow, adipocytes and lymph nodes [79]. Studies of delivery via the inhalation route have been met with mixed results: one study investigating the administration of ‘naked’ AOs through the intratracheal route showed little effect on local lung tissues while demonstrating an accumulation of AO in the liver and kidney at levels comparable to intravenous injection [80]. Other studies have shown successful AO-mediated downregulation of target protein expression in the lung [81, 82], demonstrating the effective targeting of AOs to pulmonary tissue [68]. In the context of AO therapy for asthmatic airway disease, the aerosol delivery of AO has been trialled in preclinical animal models targeting the interleukin 4 receptor α [83, 84], interleukin 5 receptor α [85], p38 mitogen-activated protein kinase [86] and CD86 [87]. In these particular studies, AO-induced knockdown of molecular targets resulted in reduction of airway hyper-responsiveness and pulmonary inflammation.

Different strategies that may be employed to enhance AO delivery also influence tissue distribution and biological actions of AOs, compared to the delivery of ‘free’ oligonucleotides. These methods may include the use of carrier molecules, conjugation and/or co-administration with other factors. One such strategy is to incorporate the AO into either a lipid [88, 89] or polymeric [90] nanocarrier to promote cellular uptake and facilitate endosomal release. Lipid nanocarriers, for in vivo use, have a surface coating (such as polyethylene glycol) that reduces the recognition and uptake of the nanocarrier by macrophages [91] while cationic lipids in the particle enable AOs to escape from the endosome after endocytosis into the target cell. However, toxicities attributed to the effect of cationic lipids on cell membranes raise doubts about the clinical use of lipid nanocarriers [92]. Typically, both lipid and polymeric nanoparticles have limited biodistribution due to their relatively large size (~ 100 nm), raising further concerns regarding toxicity [92].

Another strategy is to modify the AO by conjugation to particular ligands such as lipids [93], carbohydrates, peptides [94] or aptamers [95]. The biodistribution of conjugated oligonucleotides is less restricted than those complexed to nanocarriers, and the conjugates can easily pass across the capillary endothelial barrier. Likewise, the potential for toxicity seems to be lower than for nanocarriers [90]. Conjugated oligonucleotides also have the potential for selective targeting to a wide range of specific tissue receptors such as integrins, toll-like receptors or receptor tyrosine kinases [92]. Conjugation with *N*-acetylgalactosamine has been widely used to enhance delivery of oligonucleotides to hepatocytes [15]. However, unlike nanocarriers, conjugates are subject to rapid renal clearance due to their small size, limiting their bioavailability.

Delivery efficiency may also be improved through the co-administration of particular factors with the AO. In a study in mice, AO-induced transgene expression was improved when a specific 2′-*O*-methyl RNA was injected intramuscularly together with a non-ionic block copolymer, F127 [96]. Additionally, studies in the DMD *mdx* mouse model demonstrate that cellular uptake of a PMO is enhanced by co-administration of a glucose-fructose formulation, resulting in the restoration of higher dystrophin protein levels in the skeletal muscle [97].

Not only does an AO’s therapeutic effect depend on reaching the target cell, the AO must also reach intracellular molecular targets. Internalisation of AOs at the cell surface can occur through multiple pathways and may depend on the cell type and the cell’s physiological state [98]. However, endocytosis of the AO does not guarantee delivery to intracellular target sites as they must first escape from membrane-bound intracellular compartments [92]. Endosome contents, including internalised AOs, are largely directed to lysosomes for degradation or to the plasma membrane for expulsion to the cell exterior where they are unable to fulfil their therapeutic function. It is widely regarded that the endosomal escape barrier is one of the most significant obstacles to the effective use of AOs in therapeutics [92].

## Conclusions

Although rare in the paediatric population, lung disease associated with genetic conditions results in significant patient morbidity and mortality. Current therapies may benefit subsets of patients, but there is a need to generate new treatment strategies for specific patient populations. While AO therapy is an area yet to be explored in CF or DSPM, AOs offer potential strategies to develop targeted personalised treatments for people with specific gene mutations. Challenges remain with respect to clinical trial design in this area, due to limited cohorts of patients from which to draw participants, further compounded by stratification according to individual mutations [8, 9]. Furthermore, the variability in the natural history of disease between patients is significant, making the application of standardised outcome measures and selection of clinical endpoints difficult. One advantage of any hypothesis-driven translational research that aims to alter targeted gene expression is the ability to undertake molecular analyses that confirm changes in mRNA transcripts (reverse transcriptase PCR) or protein levels (western blotting, immunohistochemical staining or functional testing). If no changes in the RNA or protein were detected, one would not expect to see any clinical improvements. Conversely, even low changes in the RNA and induced protein have conferred substantial clinical benefits in DMD, indicating that even modest levels of functional protein can confer a therapeutic benefit. Ultimately, the investigation into relevant molecular targets, prospective AO drugs and appropriate in vivo drug delivery systems will prove vital to determine the clinical potential of this therapy to treat lung diseases of genetic origin.

## Abbreviations

508: p.Phe508del
ABCA3: ATP-binding cassette subfamily A member 3
AIDS: Acquired immune deficiency syndrome
AO: Antisense oligonucleotide
BMD: Becker muscular dystrophy
CF: Cystic fibrosis
CFTR: Cystic fibrosis transmembrane conductance regulator
DMD: Duchenne muscular dystrophy
DSPM: Disorders of surfactant protein metabolism
FDA: Food and drug administration
HCMV: Human cytomegalovirus
HIV: Human immunodeficiency virus
LNA: Locked nucleic acids
MF: Minimal CFTR function mutation
PMO: Phosphorodiamidate morpholino oligomer
PNA: Peptide nucleic acid
RF: Residual CFTR function mutation
SMA: Spinal muscular atrophy
SMN: Survival motor neuron
SP-B: Surfactant protein B
SP-C: Surfactant protein C
VEGF: Vascular endothelial growth factor
